# Parameterization of the Stomatal Component of the DO_3_SE Model for Mediterranean Evergreen Broadleaf Species

**DOI:** 10.1100/tsw.2007.27

**Published:** 2007-03-21

**Authors:** Roccío Alonso, Susana Elvira, María J. Sanz, Lisa Emberson, Benjamín S. Gimeno

**Affiliations:** ^1^ Ecotoxicology of Air Pollution, CIEMAT, Avda. Complutense 22, 28040 Madrid, Spain; ^2^ Fundación CEAM, Charles Darwin 14, 46980 Paterna, Valencia, Spain; ^3^ Environment Department, University of York, YO 10 5DD York, UK

**Keywords:** ozone critical levels, ozone flux, Mediterranean evergreen forest, Holm oak, stomatal conductance

## Abstract

An ozone (O_3_) deposition model (DO_3_SE) is currently used in Europe to define the areas where O_3_ concentrations lead to absorbed O_3_ doses that exceed the flux-based critical levels above which phytotoxic effects would be likely recorded. This mapping exercise relies mostly on the accurate estimation of O_3_ flux through plant stomata. However, the present parameterization of the modulation of stomatal conductance (g_s_) behavior by different environmental variables needs further adjustment if O_3_ phytotoxicity is to be assessed accurately at regional or continental scales. A new parameterization of the model is proposed for Holm oak (Quercus ilex), a tree species that has been selected as a surrogate for all Mediterranean evergreen broadleaf species. This parameterization was based on a literature review, and was calibrated and validated using experimentally measured data of g_s_ and several atmospheric and soil parameters recorded at three sites of the Iberian Peninsula experiencing long summer drought, and very cold and dry winter air (El Pardo and Miraflores) or milder conditions (Tietar). A fairly good agreement was found between modeled and measured data (R^2^ = 0.64) at Tietar. However, a reasonable performance (R^2^ = 0.47–0.62) of the model was only achieved at the most continental sites when g_s_ and soil moisture deficit relationships were considered. The influence of root depth on g_s_ estimation is discussed and recommendations are made to build up separate parameterizations for continental and marine-influenced Holm oak sites in the future.

## INTRODUCTION

Ozone ($O_3$) critical levels (CLs) have been established in Europe under the framework of the Convention on Long-Range Transboundary Air Pollution (CLRTAP) to assess risks of $O_3$ damage on different plant receptors at regional and continental scales, and to formulate the most suitable policy to reduce those risks. Ozone CLs for forest trees were previously based on the AOT40 exposure index (accumulated exposure above a cut-off of 40 nl $l^{-1}$ when the concentration exceeds 40 nl $l^{-1}$ during daylight hours over a stated time period). New CLs based on $O_3$ stomatal flux have been recently proposed[1,2] as it is widely recognized that $O_3$ phytotoxic effects are closely related to the amount of pollutant entering the plant through the stomatal pores and reaching the sites of damage within the leaves[3].

The EMEP (European Monitoring and Evaluation Programme) of the CLRTAP is now using an $O_3$ deposition module ($DO_3SE$, Deposition of Ozone and Stomatal Exchange)[4] to help in defining the areas where exceedances of $O_3$ $CL_s$ and areas with high ozone fluxes would occur. This module incorporates a stomatal conductance ($g_s$) model to calculate $O_3$ stomatal fluxes for different vegetation types. The model uses the multiplicative approach defined by Jarvis[5] and modified by Emberson et al.[6]:

$$g_s = g_{max} * f_{light} * \max\{f_{min}, (f_{phen} * f_{temp} * f_{VPD} * f_{SWP})\}$$

where $g_s$ for a given species is calculated as a function of the maximum $g_s$ value for that particular species ($g_{max}$), and modified according to growth stage ($f_{phen}$) and prevailing environmental factors that include photosynthetic active radiation ($f_{light}$), temperature ($f_{temp}$), air vapor pressure deficit ($f_{VPD}$), and soil water potential ($f_{SWP}$). $f_{min}$ is the relative minimum stomatal conductance that occurs during daylight hours.

The parameterization of this model should adequately reflect the stomatal behavior of the different receptors before accurate $O_3$ risk assessments could be performed in the European region. Therefore, functions representing the relationships between $g_s$ and environmental variables, such as VPD, soil moisture deficit (SMD), and plant phenology, need to be adjusted for Mediterranean tree species.

Holm oak (*Quercus ilex*) has been proposed in this context to be used as a surrogate for all Mediterranean evergreen broadleaf species due to its wide distribution across the region. The aim of this paper is to propose a new parameterization of the $DO_3SE$ $g_s$ model for this species and to evaluate the performance of the reparameterized model using field $g_s$ measurements under a wide range of Mediterranean environments.

## MATERIAL AND METHODS

A parameterization of the $DO_3SE$ $g_s$ model for Mediterranean broadleaf forests was previously proposed by Emberson et al.[7] and revised in Emberson et al.[8] based on the information available from scientific literature. Recently, published studies were pooled together with the dataset previously considered to propose a new parameterization for Holm oak. This new literature-based parameterization was tested against measurements at leaf level on adult Holm oak trees (*Q. ilex* subsp. *ballota*)[Desf.] Samp) growing under different environmental conditions. Measurements were made at three different sites in Spain: El Pardo (40º 30´5 N, 3º 45´16 W) and Miraflores de la Sierra (40º 48´ N, 3º 48´ W) located in the Madrid region in the central area of the Iberian Peninsula, and Majadas del Tietar (Cáceres, 39º 56´58.4" N, 5º47´17.2" W) in the western area. The three sites present a continental Mediterranean climate (Table 1) with long summer drought periods whose duration and magnitude depend on interannual climatic fluctuations. Winter temperatures are colder and VPD is higher in central Spain (El Pardo and Miraflores) than in Tietar due to the higher elevation and the lack of oceanic influence.

Stomatal conductance to water vapor ($g_{s\ H2O}$) was measured using a LICOR-6400 infrared gas analysis system (LiCor Inc., Lincoln, NE, USA). Measurements were performed under varying environmental conditions of light, temperature, and air relative humidity. Six to ten trees were sampled per site. Daily profiles of $g_{s\ H2O}$ rates were seasonally measured on three current- and three previous-year leaves per tree in El Pardo and Miraflores for 2 years (June and September 2004; February, June, and October 2005; March 2006). Five previous-year leaves per tree were measured at Majadas del Tietar from sunrise to midday (December 2003; February, May, June, July, August, and October 2005). Gas exchange rates were expressed on a projected

**TABLE1**
**Environmental Conditions at the Three Experimental Sites**

| | Elevation (m a.s.l.) | Rainfall (mm) | Temp (ºC) | T. summer (ºC) | T. winter (ºC) |
|---|---|---|---|---|---|
| El Pardo | 610 | 522 | 14 | 22 | 4 |
| Miraflores | 1059 | 880 | 12 | 19 | 3 |
| Tietar | 258 | 572 | 17 | 26 | 9 |

leaf area basis. Predawn leaf water potential (LWP) was measured with a Scholander-type pressure chamber (SKMP 1400, Skye Instruments Ltd., U.K.) on three to four branches per tree. Soil water content (SWC) at Majadas del Tietar was measured with a Delta-T Theta Probes (Skye Instruments Ltd., U.K.) at depths of 17, 37, and 57 cm.

Physiological data obtained during the first year of measurements at El Pardo and Miraflores were used to calibrate the $g_s$ model. Photosynthetic active radiation (PAR), air temperature, and VPD data measured with the LICOR-6400 were used for function adjustments of the model. The resulting model was validated using the second year of measurements at El Pardo and Miraflores, and the dataset measured at Tietar. Simple regression analyses were used to study the relationships between measured and calculated data. Alpha was set at 0.05 for all comparisons. All the analyses were performed using Statistica v5.1. (StatSoft, Inc.).

## RESULTS AND DISCUSSION

### Parameterization of the Stomatal Conductance Model for Holm Oak

The proposed parameterization of the $g_s$ model for Holm oak and the references supporting it are presented in Table 2. The greatest deviations from the original default parameterization of the model are related to $f_{phen}$, $f_{VPD}$, and $f_{SWP}$ functions. The new $f_{phen}$ function determines a seasonal depletion of Holm oak $g_s$ values during summer, while the default function expected increasing values during this season. Summer depletion has been widely documented in Mediterranean plants[26] and although it is mainly related to drought stress, it has also been described in plants growing under well-watered conditions. Therefore, the new $f_{phen}$ function could be used potentially as a surrogate of $g_s$ and mild soil moisture deficit relationships. The new $f_{VPD}$ function expands the minimum VPD value initially considered in the default $g_s$ model from 3.2 (1.6 for Mediterranean conifers) to 4 kPa, in addition the VPD limitation to $g_s$ is not expected to occur until plants experience VPD values greater than 2.2 kPa. This function would then account for the adaptation of Holm oak to the high VPD values commonly experienced in the Mediterranean area and also for the depletion in $g_s$ levels during summer central hours of the day in association with high VPD values[26,27]. In regards to the influence of SMD on $g_s$, soil moisture deficit is not being considered to limit $O_3$ uptake ($f_{SWP}$ = 1) when only large-scale risk assessment is intended, since the most sensitive conditions are assumed[2]. However, if more realistic estimations of $O_3$-induced effects are considered, an $f_{SWP}$ or $f_{SMD}$ function should be included though this would have to be formulated so as to complement the $f_{phen}$ function that currently allows for mild soil water deficits. The proposed $f_{SMD}$ was derived from the compilation of peer-reviewed published data concerning Holm oak $g_s$

relationships with varying soil or predawn LWP conditions. According to this function, a steady decrease in $g_s$ values would be expected when LWP or SWP range between –0.2 and –4.5 MPa (Table 2).

**TABLE 2**
**Reparameterization of the $DO_3SE$ $g_s$ Model Based on Literature Review***

| | | *Q. ilex* | References |
|---|---|---|---|
| $g_{max}$ | | 285 | [9,10,11,12,13,14,15,16,17,18,19,20,21] |
| $f_{min}$ | | 0.02 | [12,14] |
| $f_{phen}$ | SGS | 0 | [9,13,22] |
| | EGS | 365 | |
| | $A_{start}$ | 20 Mar (78) | |
| | $A_{end}$ | 15 Nov (320) | |
| | $f_{phen}$ a | 1 | |
| | $f_{phen}$ b | 1 | |
| | $f_{phen}$ c | 0.3 | |
| | $f_{phen}$ d | 1 | |
| | $f_{phen}$ S | 130 | |
| | $f_{phen}$ E | 60 | |
| $f_{light}$ | α | 0.009 | 13 |
| $f_{temp}$ | $T_{min}$ | 2 | [10,15,19,21,23] |
| | $T_{opt}$ | 23 | |
| | $T_{max}$ | 38 | |
| $f_{VPD}$ | $VPD_{max}$ | 2.2 | [10,12,13,15] |
| | $VPD_{min}$ | 4.0 | |
| $f_{SWP}$, $f_{SMD}$ | $SWP_{max}$ | $f_{SWP}$ = 1 (–0.2) | [12,13,16,24,25] |
| | $SWP_{min}$ | $f_{SWP}$ = 1 (–4.5) | |

* $g_{max}$ is the maximum $g_s$ expressed as mmol $H_2O$ $m^{-2}$ $s^{-1}$. SGS, start of growing season; EGS, end of growing season; $A_{start}$, day when $f_{phen}$ starts decreasing; $A_{end}$, day when $f_{phen}$ reaches maxima values again; a, $f_{phen}$ when SGS < dd < $A_{start}$; b, $f_{phen}$ value when $f_{phen}$ starts decreasing; c, minimum $f_{phen}$ value; d, $f_{phen}$ value when $f_{phen}$ ends increasing; S, number of days during the decline of $f_{phen}$ to reach its minimum; E, number of days for $f_{phen}$ from minimum to reach its maximum. Temperatures minima, optima, and maxima are expressed in ºC. VPD values are in kPa. SWP values are in MPa. Functions $f_{light}$, $f_{temp}$, $f_{VPD}$, and $f_{SWP}$ published in UNECE,2004[1].

## Calibration of the Reparameterized Model Using El Pardo and Miraflores Data (2004–2005)

The calibration of the reparameterized model was carried out using the data collected in El Pardo and Miraflores from June 2004 to February 2005. When no $f_{SMD}$ was considered, the performance of the reparameterized model was somewhat poor ($R^2$ = 0.27) when measured and calculated data were compared (Fig. 1), overestimating low $g_s$ values and underestimating high values. However, a better fit was found when soil moisture deficit was included in the analysis ($R^2$ = 0.62). Since no SWP data were available at these sites, a soil moisture deficit function ($f_{SMD}$) was adjusted using predawn LWP values. The proposed $f_{SMD}$ based on literature data fitted well with the actual $g_s$ and predawn LWP collected at both sites. The performance of the reparameterized model was highly dependent on the consideration of this function. However, some underestimation of high $g_s$ was still observed due to the $f_{phen}$ that predicts maxima $g_s$ values during winter, while oak trees at these sites presented the highest $g_s$ in spring when air temperature and humidity became optima. Further adjustments of $f_{light}$, $f_{temp}$, or $f_{VPD}$ to experimental data did not result in significant improvement on the model performance.

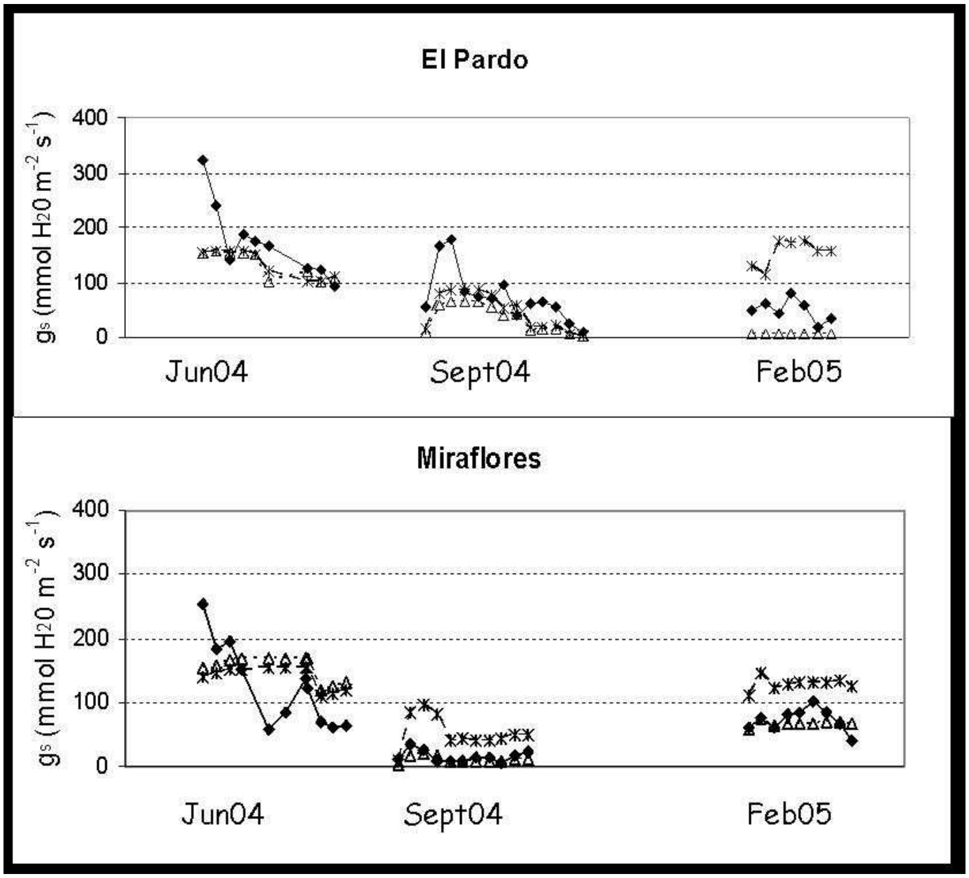

**FIGURE 1**. Calibration of the $g_s$ model for *Q. ilex* using measured data at El Pardo and Miraflores during the period June 2004 to February 2005. ♦, measured data; ∗, calculated data based on literature review parameterization; Δ calculated data based on literature review parameterization including $f_{SMD}$ function.

An in-depth analysis of late-summer data (September 2004; period of maximum drought stress) recorded at both sites indicated that the inclusion of $f_{SMD}$ was less effective in El Pardo than in Miraflores. Although air temperature and VPD values were slightly higher in El Pardo than in Miraflores, measured $g_s$ data and predawn LWP were also higher, suggesting that trees in El Pardo were less drought stressed than in Miraflores. The greater soil depth in El Pardo enables roots to grow and penetrate more in the soil profile, accessing subterranean water and detaching plant water relationships from superficial soil moisture. In this case, including an $f_{SMD}$ function only slightly improved model adjustment to measured data. However, calculated $g_s$ of trees growing under strong drought stress conditions in Miraflores required alteration of the $f_{SMD}$ function for better adjustment to measured values. These results indicate the need of considering drought stress conditions when modeling $O_3$ fluxes into Mediterranean vegetation if appropriate estimates of the risks associated to this pollutant are to be achieved as SMD represents a major constraint for plant performance in this area.

## Validation of the Reparameterized Model

The validation of the reparameterized model involved the use of three independent datasets recorded at El Pardo and Miraflores (June 2005 to March 2006) and Tietar (December 2003 to October 2005). The model was run excluding and including the $f_{SMD}$ function. The reparameterized $DO_3SE$ $g_s$ model performed differently at the three sites and the inclusion of the $f_{SMD}$ was especially important in those sites where trees experience strong drought stress (Fig. 2). The performance of the proposed $f_{SMD}$ proved to be adequate since the actual $g_s$ and LWP data recorded at the Tietar site fitted well with it. Moreover, this function shows potential to be used as a $f_{SMD}$ function since a significant correlation between LWP and SWC was found at a depth of 57 cm ($R^2 = 0.75$); lower correlation was found when SWC at 17 or 37 cm were considered showing $R^2$ values of 0.64 and 0.66, respectively. These results suggest that the hydrological status of Mediterranean evergreen trees is best associated with SWC at great soil depths. In fact, Holm oak roots can reach soil depths up to 5 m with a root length density almost uniform with depth[28], enabling trees to uptake subterranean water and decoupling water availability from surface soil moisture, local precipitation, and air temperature. A new SMD module of the $DO_3SE$ model is currently being developed[8] where root zone moisture is calculated considering atmospheric variables, such as precipitation, temperature, and VPD, together with canopy transpiration and root depth. However, access to the subterranean water table is more difficult to estimate since other regional environmental conditions need to be considered. This is an issue that might require further development, especially under continental conditions.

The calculated and measured $g_s$ values matched well with measured values in Tietar, the site experiencing the mildest winter air temperatures and highest air relative humidity, regardless of the inclusion of the $f_{SMD}$ function (see Table 3 and Fig. 2). However, the model only performed reasonably well at the two continental sites showing the most extreme winter conditions (El Pardo and Miraflores) when the $f_{SMD}$ function was considered (see Table 3). The better performance of the model at Tietar than at the other two sites appears to be related to the fact that $f_{phen}$ predicts the greatest $g_s$ levels in late winter, a condition that may only be fulfilled at marine-influenced sites where winter temperatures and VPD are mild, but not at Mediterranean continental sites where very cold and dry winter air represent a major constraint for stomatal opening during this season. These results support the need of constructing two different $f_{phen}$ parameterizations for Holm oak to account for the more restrictive environmental conditions experienced by this species at continental than at marine-influenced sites. Further efforts should be made in the future in this regard including comparison with the other Holm oak subspecies existing in the Iberian Peninsula (*Q. ilex* subsp. i*lex*).

## CONCLUSIONS

The reparameterization of the $DO_3SE$ $g_s$ model resulted in a significant improvement in the performance of the model for Holm oak, especially at the marine-influenced site. In the continental sites where trees experience strong drought stress, the inclusion of an $f_{SMD}$ function was needed to account for the related $g_s$ variations. Interestingly, tree water status can be decoupled from soil water content at the superficial soil layers depending on soil and root depth and access to subterranean water, suggesting that soil depth and characteristics of the root system should be considered in the $f_{SMD}$ function. Also, two different parameterizations of the $f_{phen}$ will be needed in order to achieve the best performance when considering continental or marine-influenced Holm oak stands. This function showed the potential to be used as a surrogate for soil water potential–related variations of $g_s$, only under moderate drought stress conditions.

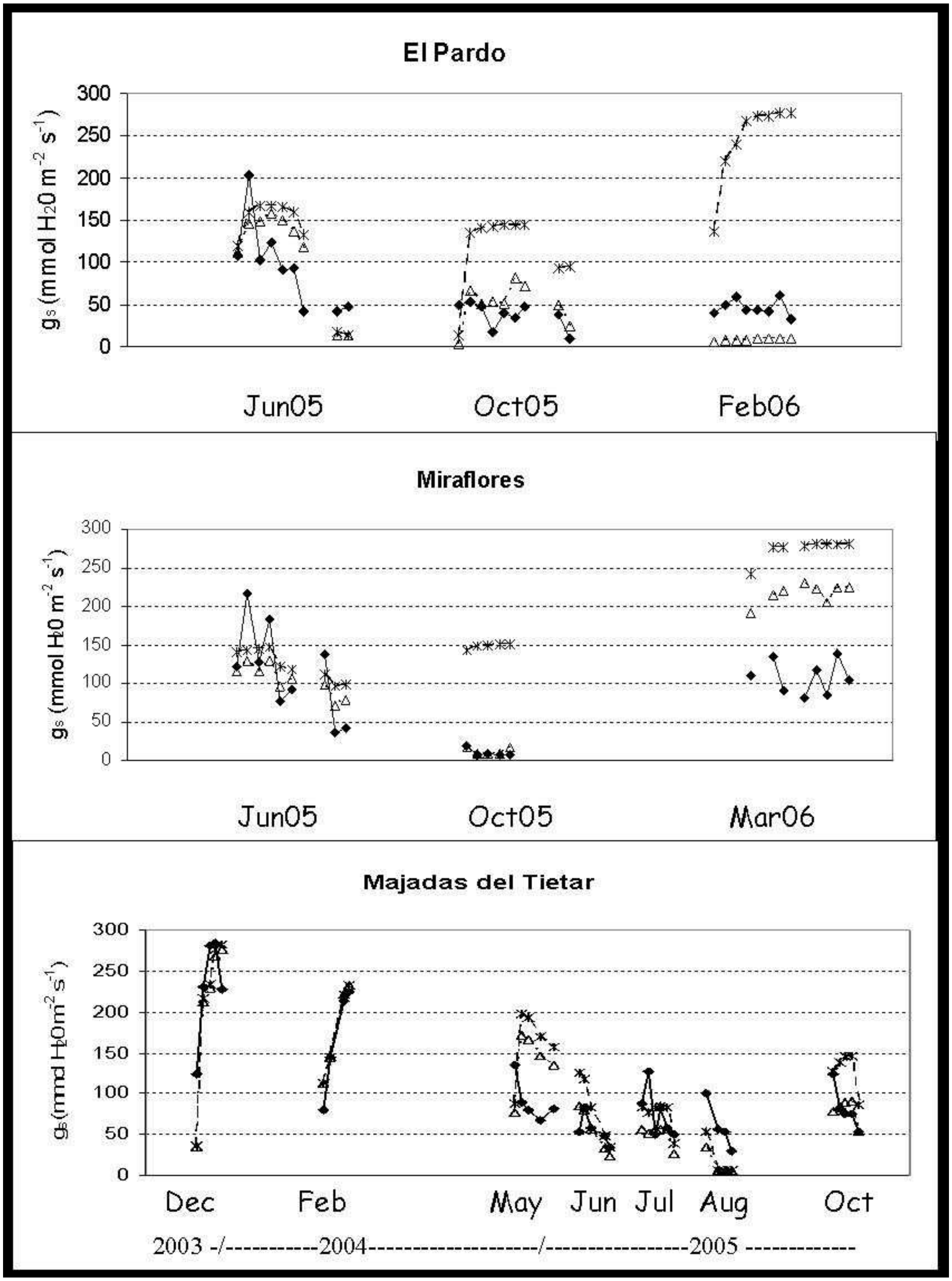

**FIGURE 2.** Validation of the $g_s$ model for *Q. ilex* using measured data at El Pardo and Miraflores during the period June 2005 through March 2006 and at Majadas del Tietar December 2003 through October 2005. ♦, measured data; ∗, calculated data based on literature review parameterization; Δ, calculated data based on literature review parameterization including $f_{SMD}$ function.

**TABLE 3**
**Results of the Regression Analyses Between Measured (M) and Calculated (C) $g_s$ Values Using the New Parameterization of the $g_s$ Model With or Without $f_{SMD}$**

| | Literature Parameterization | Literature Parameterization + $f_{SMD}$ |
|---|---|---|
| El Pardo + Miraflores 2004–2005 | C = 0.4M + 72.26; $R^2$ = 0.27 | C = 0.68M + 11.64; $R^2$ = 0.62 |
| El Pardo + Miraflores 2005–2006 | C = 0.3M + 150.85; $R^2$ = 0.04 | C = 1.13M + 14.27; $R^2$ = 0.47 |
| Majadas del Tietar 2003–2005 | C = 0.97M + 4.57; $R^2$ = 0.60 | C = 0.92M + 0.64; $R^2$ = 0.64 |

## ACKNOWLEDGMENTS

This research was carried out under the framework of the ICP-Vegetation. R. Alonso, S. Elvira, and B.S. Gimeno involvement was funded by a Spanish Ministry of Environment-CIEMAT agreement on “Critical loads and levels” and by the GALOPA project (Fundación BBVA, Spain). M.J. Sanz thanks the DGCN (MMA), the EU projects MIND and CARBOERUROPE IP for funding the projects at Majadas del Tietar, and to Generalitat Valenciana and Bancaixa for continuous support to Fundación CEAM. Additional support for this work was provided by contract SPU24 from the U.K. Department of the Environment, Food and Rural Affairs.
